# An Alternative Framework for Analyzing Financial Protection in Health

**DOI:** 10.1371/journal.pmed.1001294

**Published:** 2012-08-21

**Authors:** Jennifer Prah Ruger

**Affiliations:** Yale Schools of Medicine and Public Health, New Haven, Connecticut, United States of America

## Abstract

Jennifer Prah Ruger argues for a more multidemensional assessment of financial protection in health, which can better capture health expenditures and the full burden of health cost burdens.

**Summary Points**

- Inadequate financial protection in health increases people's vulnerability and diminishes well-being, exacerbating inequities and raising moral concerns.
- Conventional indicators of financial protection such as catastrophic spending and impoverishing spending are too narrowly conceived and likely to underestimate the adverse effects of insufficient financial protection.
- Limitations of conventional indicators include failure to capture cost barriers to access, differences in health care utilization by ability to pay, different degrees of financial protection and coverage, “informal” treatment payments, debt financing of health spending, reduced consumption of other household necessities, as well as indirect costs of illness and coping strategies.
- A multidimensional financial protection profile can capture interrelated aspects of health expenditure, such as direct and indirect costs of illness, coping strategies used to meet costs, insurance status and utilization, household consumption patterns, and how health costs affect them.
- With the data the profile yields, researchers can further study health costs' effects by poverty or income level and type of health treatment for a fuller, more comprehensive view of health cost burdens and their distribution.

## Introduction

A greater focus on the role of health systems in health, development, and economic growth has led health policy research and analysis, domestic and global, to scrutinize health financing, insurance, and financial protection. Two *World Health Reports* (2000 and 2010) [1],[2] have called for evaluating health system performance in terms of health financing, and the World Health Organization's (WHO) 64th World Health Assembly reiterated the need for sustainable health financing and universal coverage worldwide [3]. With this increased focus has come closer examination of conventional frameworks and measures of financial protection in health both from academic [4] and policy [5] circles.

Consensus had developed among academic and policy analysts on two primary metrics, catastrophic and impoverishing spending, for financial protection. Both methods use as a measure the percentage of out-of-pocket health spending in households' overall spending. They differ in the way medical spending is deemed problematic: catastrophic spending is above a threshold percentage, while impoverishing spending pushes a household below the poverty line. Both metrics are helpful indicators of the absolute and relative level of household out-of-pocket health care spending and have been employed in multiple studies worldwide [6]–[10]. Our research group conducted a study focusing on a modification of these metrics—the out-of-pocket spending burden ratio using household equivalent income derived from the Organisation for Economic Co-operation and Development (OECD) Equivalence Scale [11].

But the consensus has given way, and critiques of the conventional approach now run wide and deep. Critics include those who are most invested and who have employed these methodologies [5],[7], and those who argue that estimates of household health expenditures themselves are subject to considerable variability depending on survey design [12]. This article proposes a multidimensional financial protection profile that offers a more holistic view of health spending, one that goes beyond the level of spending to cover aspects directly related to health care, such as health care access and insurance utilization, and examines broader impacts on current and longer-term household consumption. This multidimensional approach aims to help policy makers understand the larger context of household health spending and make health and social policy adjustments to mitigate damaging effects.

## Critiques: Financial Protection Too Narrow

A recent article [4] in *PLOS Medicine* underscored numerous criticisms of the two conventional financial protection indicators. Concerns include the failure to capture the following: cost barriers to access [2],[13]–[15]; differences in health care utilization by ability to pay [16]; protection inadequacies for poor individuals [17]; measures of illness vulnerability, such as the number of chronic conditions [11]; degrees of financial protection and coverage (underinsurance) [16]; “informal” treatment payments [11]; debt or credit financing of health care expenditures [18]; and reduced consumption of other household necessities (e.g., food, education, or utilities). Also neglected are the indirect costs of illness (income loss due to poor health, for example) and strategies of coping with direct and indirect costs of illness, which themselves are costs in current or future consumption or savings. Conventional methods are likely to underestimate adverse consequences of inadequate financial protection in health.

Most damaging of the critiques is the charge that the current approach, by its inadequate representation of risk protection and of costs, can potentially mislead policy makers who, by relying on these conventional measures, might come up with misinformed policy prescriptions [4].

In a previous study, we sought to address one of these criticisms by assessing out-of-pocket spending among those with chronic illnesses as opposed to those without such conditions [11], finding that individuals with low income and multiple chronic conditions are especially vulnerable to high out-of-pocket health spending. However, this study offered only incremental expansions of conventional methodologies, which are simply too narrow to capture fully the detrimental financial consequences of health needs. We need a broader, multidimensional framework.

## Theoretical Foundations of Health Insurance and Financial Protection

Developing a framework for analyzing health insurance and financial protection requires a grasp of underlying theoretical foundations. Health insurance creates important conditions for human flourishing by, first, keeping people healthy, and second, protecting ill individuals and their households from insecurity and harmful deprivations in essential goods (e.g., food, basic education, utilities) [19]. Conventional measures of financial protection address neither of these key ethical goals adequately. A lack of access to insurance-provided financial protection increases vulnerability, undermines well-being, and hinders human flourishing.

To understand what a more complete analytical framework might look like, it is necessary first to assess what individuals and households without health insurance must do. They must forgo necessary health care, use informal risk-sharing arrangements, self-insure, drain savings, diversify assets, borrow, sell assets, and more, all of which diminish current welfare and future prospects. These funding methods, along with interrupted insurance, user fees, user charges, co-payments, deductibles, and waiting periods, fail to provide sufficient protection and deprive users of high quality, medically necessary, and medically appropriate care. Unmet health needs can lead to further health declines, illness-related direct and indirect costs, even irreversible disability and death. Access to and financing of health care have inseparable equity implications.

Analyzing the financial protection issue from these theoretical foundations provides a much broader and more complete picture of relevant factors. It also exposes the harmful health and financial consequences of inadequate health insurance and financial protection, and the distribution of those consequences.

## A Multidimensional Approach

Our research group recognizes the limitations of unidimensional catastrophic and impoverishing spending measures. We have thus pursued a multidimensional approach, quantitatively assessing important elements and their interrelations from a household perspective. Table 1 maps out our approach. In a comprehensive household survey, we empirically studied dimensions of financial protection affected by health care needs (measured as episodes of illness in the past 12 months). These dimensions include health insurance's direct, health care–related effects and its social impact beyond health. Dimensions of direct effects include (i) access to health care, at what level, what type (outpatient, inpatient, self-treatment, or no treatment), and in what facility; (ii) total costs of illness (direct, indirect, and other); (iii) health insurance type; and (iv) health insurance utilization. Dimensions of social impact include (v) coping strategies (e.g., spending income or savings, relying on relatives or friends, borrowing, food reduction); and (vi) household resource reallocation among categories such as food, transportation, education, housing, utilities, farming or business equipment, construction, and interest on loans.

**Table 1:** Multidimensional approach to analysis of financial protection.

Variable	Description
**Episodes of illness**	All episodes of illness, past 12 months
**Access to care/treatment**	
Outpatient	No overnight stay in health facility required
Level 1	0–4 treatments
Level 2	5–10 treatments
Level 3	>10 treatments
Inpatient	Overnight stay in health facility
Self-treatment	Treatment not prescribed/given by health professional
No treatment	
**Health facility type**	Community health clinic
	District hospital
	Provincial/city hospital
	Central hospital
	Regional polyclinic
	Other state facility
	Private health facility
	Village health worker
	Other
**Total costs of illness**	
Direct costs of illness	Facility
	Other (medicine, supplies)
Indirect costs of illness	Unofficial fees
	Gifts
	Transportation
	Food
	Lost income
**Health insurance utilization**	
Health insurance status	Uninsured
	Underinsured
	Insured
Health insurance utilization	Uninsured
	Insured but did not use insurance
	Insured and used insurance
Health insurance scheme	
Compulsory	Formal sector employees
Voluntary	Dependents, workers not covered by compulsory scheme
Poor	Health Care Fund for the Poor (HCFP)
Meritorious	People with substantial contribution to socialist revolution
Children under 6	Children under 6
Other	Other (e.g., dependents of police/military)
Reasons for not using health insurance	Incorrect registered health facility
	Procedures too complicated
	Expenses were not high
	Patient not shown how to use insurance
	Other (e.g., forgot insurance card)
**Coping strategy**	Income
	Savings
	Relatives/friends
	Loans
	Food reduction
**Impact on household resource allocation**	
Food	Rice, produce, meat, etc.
Education	Tuition, books, room and board
Production means	Items for farming, business, trade
Housing	Mortgage, rent
Transportation	Vehicle (motorcycle), oil, gas, repairs
Health care	Treatment, medicine, gifts to health staff
Construction	Building and repair of home, business
Charity	Gifts for mourning, for community
Durable goods	Furniture, appliances
Utilities	Electricity, water, gas, phone
Daily goods	Toiletries, kitchen supplies
Social activities	Entertainment, holidays, wedding, travel
Insurance	Property, health, etc.
Gifts	Gifts for family, friends
Tobacco/alcohol	Cigarettes, liquor, etc.
Loan interest	Interest paid on loans
Other	Expenditures not listed above
**Stratification**	
Overall	All households
By income quartiles	Q1–Q4
By poverty level	Poor
	Near poor
	Non-poor
By type of service	Outpatient
	Inpatient
	Self-treatment

The financial and health implications of health needs are interrelated. For instance, coping strategies, while helpful in stabilizing certain situations in the very short term, can damage household economic and health security over time. Decreased food consumption and stress caused by economic burdens can undercut health, and poor health weakens one's ability to work, diminishing one's capacity to repay loans—especially loans with high interest rates—and to afford other expenses such as education and work equipment. Understanding these interrelations is vital to enabling and maintaining the broader conditions for human flourishing.

## Financial Protection Profile

A financial protection profile offers a more accurate picture of how individuals and households of different poverty/income levels fare across numerous dimensions when confronting a health need.

### Total Costs of Illness

When health needs arise, households cope with multiple financial challenges, in addition to direct payments to health facilities. The total costs of treatment (inpatient or outpatient) include not only direct medical costs, but also, depending on the culture and setting, indirect costs such as gifts, unofficial payments, transportation, costs of caretakers, food costs, and lost income from missed work. Conventional financial protection measures underestimate these costs.

### Coping Strategies

Conventional indicators deem expenses “catastrophic” if they add up to a given threshold of household income. An alternative approach assesses a “catastrophic” or “impoverishing” situation based on the health and economic consequences for a household, broadly conceived. For example, catastrophic payments force households to reduce consumption necessary for general well-being and economic security or to rely on loans [20]. Such health financing measures or “coping strategies” (Box 1) are often used to finance health care and to maintain economic viability following a health shock with economic ramifications.

**Box 1. Coping Strategies**

1 Income from that month
2 Savings
3 Funds from relatives or friends
4 Borrowing
  4a Amount
  4b Interest rate
5 Reduce expenditures on food
  5a Amount reduced
  5b Duration of reduced expenditure
6 Reduce expenditures on clothing
7 Reduce expenditures on household items
8 Change purchase or amount spent on larger household expenses
9 Change purchase or amount spent on construction
10 Change purchase or amount spent on items for manufacturing and trade
11 Reduce gifts given to relatives and friends
12 Reduce expenditures on social activities
13 Reduce expenditures on cigarettes and alcohol
14 Reduce expenditures on education
  14a Amount reduced
  14b Number of children
  14c Expenditure reduction on supplies/books, etc.
  14d Number of children stopped school altogether
15 Increase labor
  15a Number of children age <18 need to provide manual labor or other adult work to secure income
  15b Number of people age >60 need to work to secure income who previously did not work
  15c Number of people currently working who have to work more hours
16 Sell household belongings (e.g., TV, refrigerator, fan)
17 Sell production means (e.g., farming tools, equipment)
18 Sell farmland
19 Sell home
20 Other (open-ended write-in question; please specify)

#### Coping strategies as they relate to total costs of illness (direct or indirect)

Coping strategies help deal with direct treatment costs as well as the indirect costs of health care and medicines, but these strategies themselves also incur costs. Understanding the full catastrophic or impoverishing impact requires examining the aggregate impact of all these costs, not just those for treatment.

In a study of 706 Vietnamese households, we found the five most common coping strategies to fund inpatient and outpatient treatments are using (i) income or (ii) savings, (iii) borrowing from relatives or friends, (iv) taking out loans, and (v) reducing food consumption [20]. For example, loans were more likely to fund extremely high-cost inpatient treatments (rather than low-cost treatments) for households of all poverty levels. Borrowing for outpatient treatments was more common among the poor and near-poor than the non-poor. Not only were loans frequent, but many households had to take out further loans to repay their original borrowing. A higher proportion of the poor (44%) than the non-poor or near-poor (24%) had to borrow to repay loans for inpatient treatment. Moreover, the likelihood of reducing food consumption to pay for extremely high-cost treatments was higher than for low-cost treatments. For both inpatient and outpatient treatments, the poor were more likely than the non-poor to reduce food.

### Treatment by Insurance Status

Health insurance status is more nuanced, with gradations of coverage, than the conventional insured/uninsured categorization. First, individuals can fall into at least three health insurance categories: (i) insured; (ii) uninsured; and (iii) insured but unable or unwilling to use coverage [21]. In other situations, individuals may be (i) insured; (ii) uninsured; and (iii) underinsured [16]. Second, insurance status may vary by each episode of treatment, rather than for each individual or household.

In our study of Vietnamese households, for example, the poor and near-poor were less likely to be insured than the non-poor, who also constituted the greatest proportion of the insured who used insurance (50% of non-poor, compared to 31% of poor and 20% of near-poor) [21]. The poor accounted for the greatest proportion of those who were insured but did not use insurance (50% compared to 23% for near-poor and 27% for non-poor). The insured experienced fewer days of missed work and school due to illness than the uninsured (9 days versus 25 days for the uninsured for inpatient treatment).

### Household Consumption Patterns

Household consumption items range from food, education, housing and health care to social activities, charity, and interest paid on loans (Table 1). In our study of Vietnamese households, compared to households without inpatient treatment, households with inpatient treatment reduced consumption of food, education and production means, and the most significant decrease occurred in the lowest income quartile of the population (Nguyen KT, Khuat OTH, Ma S, Pham DC, Khuat GTH, et al., unpublished data). Higher income quartiles showed decreases in different categories of consumption, such as durable goods. Consumption of food, education, and construction decreased for households with the most episodes of outpatient treatment, compared to households with the fewest episodes; the lowest income quartile reported the greatest food reduction. No income quartile with inpatient or high outpatient treatment costs was exempt from decreases in consumption.

## Conclusion

**Figure 1:**
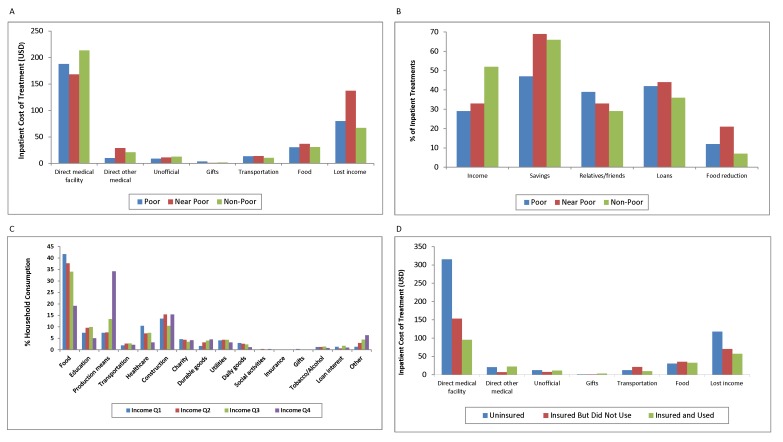
Financial protection profile. (A) Inpatient treatment costs by poverty level. (B) Inpatient treatment coping strategies. (C) Household consumption by income quartiles. (D) Inpatient treatment costs by insurance status. Note: More than one coping strategy may be used to finance health treatment; panel (B) shows the percentage of inpatient treatments involving each coping strategy, by poverty level.

In response to health expenses, households (especially the poor) may reduce essential consumption—further diminishing their economic resources—and become vulnerable to downward debt spirals. Conventional, single-measure indicators of financial protection do not capture the full breadth of health costs, nor do they illuminate how costs affect health care access and utilization. Constructing a multidimensional financial protection profile has its challenges, however. It is necessarily more data-intensive. Although some of the relevant data may be available through regularly conducted national household surveys, researchers will need to undertake original data collection; a questionnaire like ours could be integrated into national household surveys. The problems of recall error and bias affect retrospectively collected data, but survey design can mitigate them. A multidimensional profile is worth the extra effort, as it can give a more comprehensive view of illness costs, coping strategies, treatment by insurance status, and household consumption patterns (Figure 1). It presents more fully the impact of health costs, highlighting the urgent need for financial protection and offering better guidance to policy makers.

## Abbreviations

WHO: World Health Organization
